# Supplementary material for: Comprehensive Annotation and Functional Exploration of MicroRNAs in Lettuce
Source: Front Plant Sci. 2021 Dec 24;12:781836. doi: 10.3389/fpls.2021.781836 (PMC8739914; doi:10.3389/fpls.2021.781836)

T=Lsat_1_v5_gn_8_27040.1_Q=Lsa−miR6111_S=688

category=1_p=0.0207725588879266

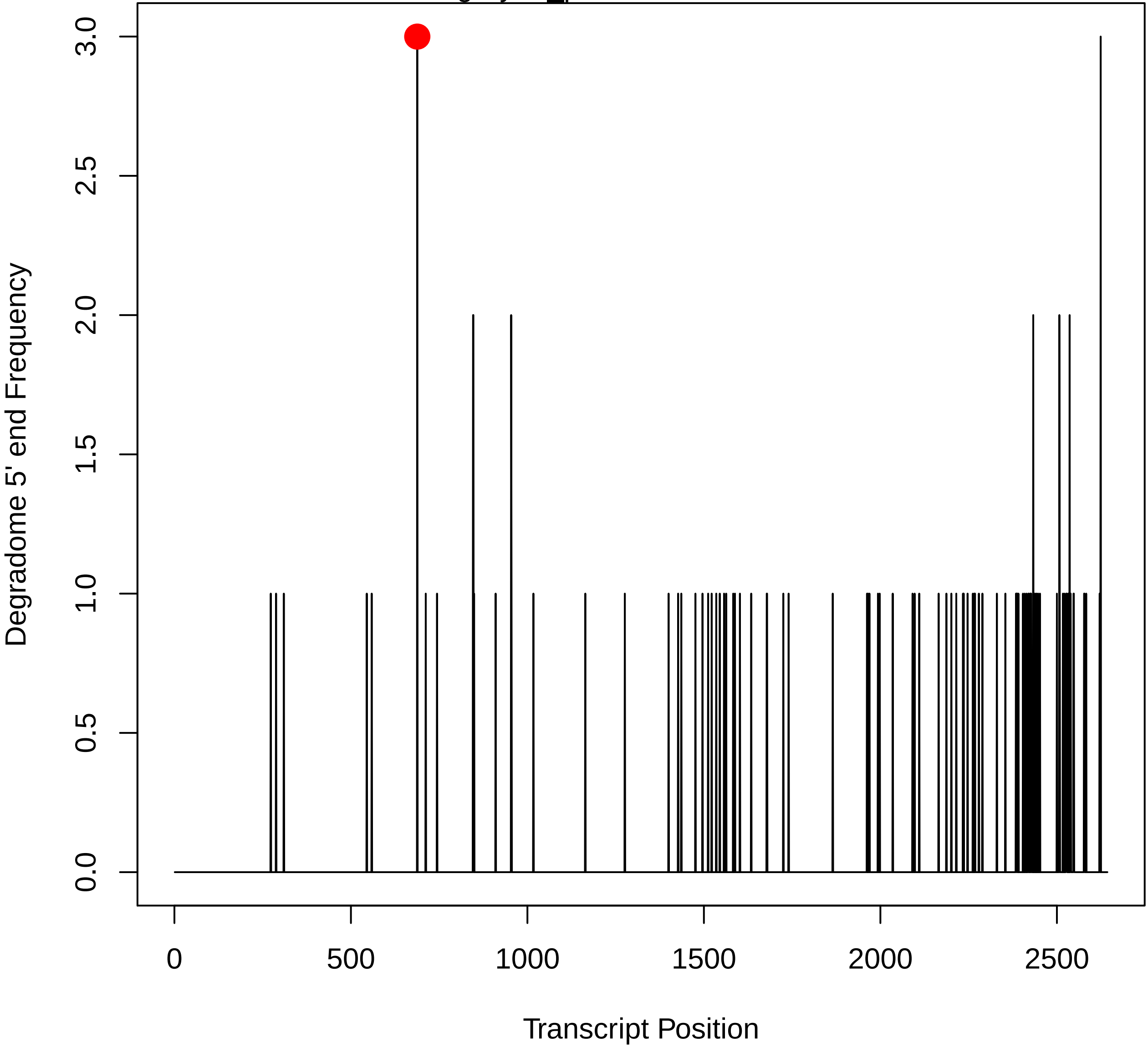

Supplement: Supplementary file 2 [file Data_Sheet_8.ZIP › GSM2230750.plot/Lsa-miR6111_Lsat_1_v5_gn_8_27040.1_688_TPlot.pdf]
